# The role of maternal serumbeta-HCG and PAPP-A levels at gestational weeks 10 to 14 in the prediction of pre-eclampsia

**DOI:** 10.12669/pjms.303.4554

**Published:** 2014

**Authors:** Ozkan Ozdamar, Ismet Gun, Ugur Keskin, Necmettin Kocak, Ercument Mungen

**Affiliations:** 1 Ozkan Ozdamar, Department of Obstetrics and Gynecology, Golcuk Military Hospital, Golcuk, Kocaeli, Turkey.; 2 Ismet Gun, Associate Professor, Department of Obstetrics and Gynecology,GATA Haydarpasa Training Hospital, Istanbul, Turkey.; 3 Ugur Keskin, Assistant Professor, Department of Obstetrics and Gynecology, GATA Medical Faculty, Ankara, Turkey.; 4 Necmettin Kocak, MD, Department of Public Health, GATA Medical Faculty, Ankara, Turkey.; 5 Ercument Mungen, Professor, Department of Obstetrics and Gynecology,GATA Haydarpasa Training Hospital, Istanbul, Turkey.

**Keywords:** PAPP-A, β-hCG, First trimester, Pre-eclampsia.

## Abstract

***Objective: ***We aimed to detect whether maternal serum free β-hCG and PAPP-A levels and NT measurements vary between normal pregnancies and those that subsequently develop pre-eclampsia and to evaluate the role of these screening serum analytes in the prediction of pre-eclampsia.

***Methods: ***Using a case-control study design, we identified all women who had been screened by double test within 11+0 and 13+6 weeks of gestation and who had developed pre-eclampsia during the subsequent pregnancy course, over a 6-year period between January 2006 and December 2012 at two tertiary referral hospital. All women who had undergone a double test during that time, without a diagnosis of pre-eclampsia and who had not had any adverse obstetric outcomes, were also identified, and three women among them were randomly selected as controls for each case. Maternal and neonatal data were abstracted from the medical records and PAPP-A, β-hCG, NT and CRL MoM values were compared between the two groups.

***Results: ***Although β-hCG values show no statistically significant difference (p=0.882), PAPP-A levels were significantly reduced in the pre-eclampsia group compared to the control group (p<0.001). NT and CRL values showed no significant difference between the two groups (p=0.674 and p=0.558, respectively).

***Conclusion: ***Measuring PAPP-A in the first trimester may be useful in the prediction of pre-eclampsia.

## INTRODUCTION

Pre-eclampsia is a heterogeneous, multisystem syndrome that accounts for 10% to 15% of maternal deaths.Although the signs and symptoms characteristically manifest in the second to third trimester, the underlying pathology may already be apparent from the very early stages of pregnancy.In preeclamptic women, insufficient placentation resulting from the impaired cytotrophoblastic invasion of the myometrial segments of the spiral arteries, results in a restricted blood supply to the fetus and placenta. As a result of this abnormal cytotrophoblastic invasion, placental ischemia is thought to develop.^1^ Given its frequent occurrence and potential morbidity and mortality, early prediction of pre-eclampsia would allow for close antenatal surveillance and preventive strategies.

First and second trimester serum screening has been used for many years as a method of identifying fetuses at increased risk of chromosomal abnormalities, in particular trisomy 21. In addition to these aneuploidies, a variety of other pregnancy outcomes, such as pre-eclampsia, have been associated with abnormal values of these screening test analytes.By numerous studies, maternal serum β-hCG elevation, which has been defined with cut-offs varying from 2.0 MoM to 4.0 MoM, has been demonstrated to be in correlation with subsequent pre-eclampsia.^2^^-^^4^ However, all those studies were conducted in the second trimester and they indicated this elevation to be between 15^th^ to 19^th^ weeks of gestation or after this period, close to the onset of the pre-eclampsia.Some other studies have been conducted during the first trimester and they concluded that there were no correlation between elevated maternal serum β-hCG levels and subsequent development of pre-eclampsia.^5^^,^^6^

The origin of the preeclampsia is believed to be lying in the placenta, hence many investigators hypothesized a relationship between the PAPP-A levels and preeclampsia. Many studies have identified an increase in adverse obstetrical outcomes associated with low levels of maternal serum PAPP-A.^6^^,^^7^First trimester serum PAPP-A levels were found to be decreased in pregnant women who were destined to develop pre-eclampsia.^7^^-^^9^

In this study, we aimed to detect whether there is a significant difference in maternal serum free β-hCG and PAPP-A levels in addition to NT measurements between normal pregnancies and those that subsequently develop pre-eclampsia and to evaluate the role of first trimester screening serum analytes in the prediction of pre-eclampsia.

## METHODS

The study was approved by the institutional review board of the hospital and regional ethical committee.We designed a bi-centered retrospective case control study. 6822 patients who had been performed double tests within 11+0 and 13+6 weeks of gestation between January 2006 and December 2012 were identified through the use of a database consisting of patients with singleton pregnancies that gave birth at the GATA Haydarpasa Training Hospital, Obstetrics and Gynecology Service, İstanbul and the GATA Hospital of Medical Faculty of Medicine, Obstetrics and Gynecology Service, Ankara. Study group was formed with patients who developed pre-eclampsia during the subsequent pregnancy course after double tests (Group 1) (n=60). Data were collected until we obtained sufficient numbers of subjects to achieve necessary power.

The pregnancies complicated by documented chromosomal abnormalities or major structural anomalies were excluded from the final study group. Patients whose records exactly could not be obtained, were also excluded. In order to construct a control group, we identified all patients who underwent double test within 11+0 and 13+6 weeks of gestation during the same study period, and who did not have pre-eclampsia and any adverse obstetric outcome (i.e. pre-term labor, gestational diabetes, fetal growth restriction, intrauterine fetal death). Among this group of women without pre-eclampsia, three controls were randomly selected for each study subject based on a random numbers table (Group 2) (n=180). Demographic information, screening test results and obstetric and perinatal outcomes, were abstracted from the hospital medical records

The criteria adopted for diagnosis of pre-eclampsia was systolic arterial blood pressure ≥140 mmHg and/or diastolic arterial blood pressure ≥90 mmHg on two occasions, separated by 6 h, with ≥300 mg/24 h proteinuria.^10^^,^^11^

Serum concentrations of β-hCG and PAPP-A were analyzed by specific immunoassay at the time of serum sampling as part of the routine screening for fetal Down’s Syndrome and all results were expressed as multiple of the median (MoM) of the expected normal median for a pregnancy of the same gestational age.Ultrasonographic assessment was carried out by two experienced obstetric sonographers at gestational weeks 11+0 to 13+6. Nuchal translucency (NT) and crown-rump length (CRL) were measured via two ultrasonography devices (Toshiba PowerVision 6000 SSA-370A, Toshiba Medical Systems Co, Ltd, Tokyo, Japan and Voluson E8 Expert, GE Healthcare, Wauwatosa, WI, USA).

Demographic properties such as maternal age at serum sampling date (years), fetal gestational age (days) at the time of inclusion, fetal gestational age (weeks) at birth, fetal body weight (g) at birth and maternal body weight (kg) at the time of inclusion were examined.Perinatal variables, including sampling date, maternal age, gestational age at both sampling and delivery, maternal weight, delivery date, fetal outcome and smoking or IVF status, were obtained from the patient database recorded and constructed by the clinic.

Two groups were compared in terms of PAPP-A, β-hCG, NT and CRL MoM values. The databank was constructed in Excel (Microsoft, New York, NY, USA), and the analyses were conducted using SPSS (Statistical Package for the Social Sciences; Chicago, IL, USA) version 15.0.Descriptive statistics were expressed as mean (± standard deviation) for demographic properties and median (range) for biochemical and sonographic measurements.Kolmogorov-Smirnov test was used to evaluate whether the continuous variables were normally distributed.When a comparison was made between two groups, Student’s t-test for symmetrical distribution and the Mann–Whitney U test for asymmetrical distribution were utilized. Specificity, sensitivity, positive predictive values and negative predictive values were calculated for different serum PAPP-A levels and receiver-operating characteristics (ROC) curves were constructed. Binary logistic regression was used for further analyses of PAPP-A values between groups. The power of this study was calculated by using G Power 3.1.3 program.Assuming the alpha error as 0.05 and beta-error as 0.20, the power of this study according to PAPP-A was calculated to be 96%.Statistical significance was defined as p <0.05.

## RESULTS

Sixty women with hypertensive disease and 180 normotensive women were identified.The demographic features of the study population are shown in Table-I. Table-I shows that the mean birth-weight was 2381.67 (± 1045.28) in the pre-eclampsia group and 3388.06 (± 358.15) in the control group (p<0.001). Mean weight at the time of serum sampling was 60.61 (± 9.212) for women in control group and 70.26 (± 12.262) for pre-eclamptic women (p<0.001). Mean gestational week at delivery was 34.9 (± 3.61) and 39.02 (± 0.865) in pre-eclampsia and control groups, respectively (p<0.001).

**Table-I:** Demographic features of patients in pre-eclampsia and control groups

	*Group 1* *(Pre-eclampsia) (n=60)*	*Group 2* *(Control) (n=180)*	*p*
	*MeanSt. Deviation*	*MeanSt. Deviation*	
Gestational age at delivery (weeks)	34.93.61	39.020.865	<0.001*
Birth-weight (g)	2381.671045.28	3388.06358.15	<0.001**
Maternal age (years)	29.305.729	28.124.329	0.238*
Gestational age at sampling (days)	86.534.188	86.734.617	0.773**
Maternal weight (kg)	70.2612.262	60.619.212	<0.001*

* Mann-Whitney U test,

** t-test

Serum concentrations of β-hCG and PAPP-A were analyzed and CRL and NT were measured at the time of routine screening for Down’s Syndrome.The median values (range) of maternal serum β-hCG MoM and PAPP-A MoM in addition to CRL MoM and NT MoM in the study population are shown in Table-II. Although β-hCG values show no statistically significant difference (Mann Whitney U test, p=0.882), PAPP-A levels were significantly reduced in the pre-eclampsia group compared to the control group (p<0.001).NT and CRL values showed no significant difference between the two groups (t-test, p=0.674 and p=0.558, respectively).Mean PAPP-A Mom levels were 1.252 in the control population and 0.920 in the pre-eclampsia group.

**Table-II:** Median values of investigated parameters of both groups

	*Group 1* *(Pre-eclampsia) (n=60)*		*Group 2* *(Control) (n=180)*		
	*Median*	*Range*	*Median*	*Range*	*p*
β-hCG	0.985	0.195-3.275	1.018	0.260-11.121	0.882*
PAPP-A	0.792	0.260-5.089	1.184	0.254-4.004	<0.001*
CRL	59.30	46.00-76.0	61.00	38.00-82.00	0.558**
NT	0.932	0.570-1.70	0.934	0.575-3.166	0.674**

* Mann-Whitney U test,

** t-test

**Fig.1:**
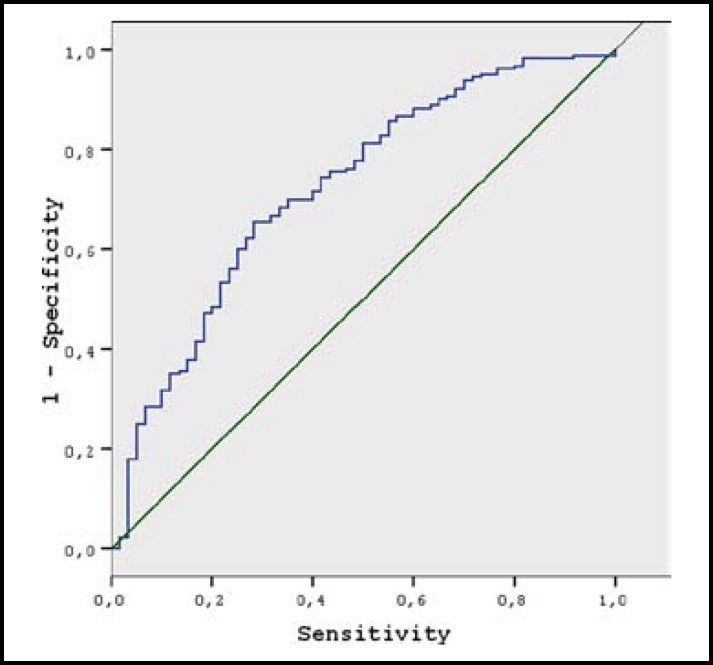
Receiver operating characteristics (ROC) curve for PAPP-A values

In ROC analysis (Fig.1), area under curve (AUC) was calculated as 0.72 (Standard Error, SE: 0.039) (95% CI 0.646 – 0.798). Specificity, sensitivity, PPV and NPV values for different PAPP-A cutoff points are shown in Table-III. In our study, the point with the highest sensitivity and specificity for maternal serum PAPP-A values to predict pre-eclampsia was determined to be 0.956. Furthermore, each unit decrease in PAPP-A values causes 3.9-fold increase in risk of developing pre-eclampsia (95% CI: 1.887 - 8.087). 

**Table-III:** Sensitivity, specificity, PPV and NPV of maternal serum PAPP-A MoM at various cut-off levels in the prediction of pre-eclampsia

**PAPP-A**	**Sensitivity**	**Specificity**	**PPV**	**NPV**
**≤0.956**	70%	65.6%	67%	68.6%
**≤ 0.90**	60%	70%	66%	63%
**≤ 0.80**	50%	78.3%	69%	61%
**≤ 0.70**	43.3%	86.7%	76%	60%
**≤ 0.60**	30%	92.8%	80%	57%
**≤ 0.50**	16.7%	98.3%	90%	52%

PPV, positive predictive value; NPV, negative predictive value.

## DISCUSSION

Since pre-eclampsia is a major contributor to maternal mortality and morbidity, reliable screening tests to predict women at risk of developing pre-eclampsia and to identify preventable causes of pre-eclampsia have been investigated.Although many biochemical markers, including tests for Down’s serum screening markers and hormones have been proposed for their relation to placental perfusion, vascular resistance, and placental products, only a few reached sensitivities and specificities above 90%.However, no single test seems to be effective in the prediction of pre-eclampsia due to the complex and heterogeneous nature of the disease.^12^

Current data suggest that there is an association between elevated maternal serum free β-hCG in the second trimester and subsequent development of adverse obstetrical outcomes, including pre-eclampsia.^2^^,^^4^^,^^13^^-^^16^ These studies have demonstrated that β-hCG levels in the maternal serum are increased in the second trimester of pregnancies that subsequently develop pre-eclampsia while many others indicated that these values show no difference compared to controls in the first trimester.^5^^,^^6^^,^^17^ Our study results for β-hCG were comparable with those of previous studies and maternal serum-free β-hCG in the first trimester was not significantly higher than those of controls in pregnancies that subsequently develop pre-eclampsia.These results indicated that free β-hCG elevation in pregnancies which subsequently develop pre-eclampsia does not occur in the first trimester.Sebire et al.^5^ concluded that maternal serum-free β-hCG concentration at 10-14 weeks, as distinct from the findings in the second trimester, was not significantly different between normotensive women and those that subsequently develop pre-eclampsia. Spencer et al.^18^ demonstrated that maternal serum-free β-hCG values at the first trimester were not significantly different from those of normal outcome group. Dugoff et al. suggested the combination of β-hCG and inhibin-A at 15-19 weeks to be useful in predicting women who were at high risk for adverse pregnancy outcomes including pre-eclampsia.^19^ In the light of these studies, we may conclude that free β-hCG values increase closer to the onset of pre-eclampsia, not in the first trimester.

Both β-hCG and PAPP-A are placental products thatare primarily produced by syncytiotrophoblasts.Elevated serum levels of β-hCG in the second trimester may reflect the impaired placental perfusion and placental damage, which is followed by spillage into the maternal circulation.However, these effects may not appear in the first trimester. In the first trimester, placental damage may be insufficient to cause an increase in serum β-hCG levels.On the other hand, altered placental perfusion and placental damage may trigger the over-production of β-hCG by syncytiotrophoblasts.^5^

PAPP-A is a large protein complex that has been shown to reduce in maternal serum at gestational weeks 11 + 0 to 13 + 6 in pregnancies subsequently developing pre-eclampsia. However, the sensitivity of screening for pre-eclampsia is poor, as only 8–23% of the affected cases have serum levels below the 5th centile, which is about 0.4 MoM. On the other hand, at the 5th centile of normal for PAPP-A the reported odds ratios for pre-eclampsia vary between 1.5 and 4.6.^6^^-^^9^^,^^20^^-^^21^ Similar to these studies, we detected significant differences in maternal serum PAPP-A concentration at 11+0 to 13+6 weeks of gestation between the normotensive pregnancies and those that subsequently develop pre-eclampsia. We determined the sensitivity as 70% for 0.956 value of PAPP-A in maternal serum. Also, each unit decrease in PAPP-A values was associated with 3.9-fold increase in risk of developing pre-eclampsia. Table-IV: comparatively shows our results and results of similar studies in the literature. The OR value we determined was in accordance with the OR values determined in previous studies. Table-IV: Majority of our pre-eclampsia cases were late-onset and the mean gestational age at delivery was 34.9 (± 3.61) in pre-eclampsia group. Table-I: For late-onset disease, PAPP-A might not be an ideal marker for subsequent pre-eclampsia.

Early detection of the onset of pre-eclampsia and the optimal time to intervene to decrease both the maternal and fetal mortality and morbidity still hold their importance.Although numerous biochemical markers have been proposed to predict women at risk to develop preeclampsia, many of them suffer from poor specificity and predictive values for routine use in clinical practice. Still, one of the most important questions remained to be answered is what the correct period for screening to predict pre-eclampsia is? In this early period of pregnancy, PAPP-A may become a promising marker for the early prediction of pre-eclampsia.However, instead of using maternal serum PAPP-A alone, combination with other serum markers or imaging tools such as first trimester uterine artery Doppler as previously published may be more valuable in early prediction. Additionally Canini et al.^22^ have suggested that PAPP-A is more useful as a marker of fetal growth restriction than of preeclampsia. Spencer et al.^21^ concluded that low levels of maternal serum PAPP-A were associated, in the absence of an abnormal karyotype, with an increased risk for subsequent delivery of an SGA infant. However a systematic review of World Health Organization indicated that there is no clinically useful screening test to predict the development of preeclampsia.^23^

**Table-IV:** Summary of various studies of PAPP-A in prediction of pre-eclampsia

*Study*	*Pre-eclampsia (n)*	*MoM*	*OR or RR*	*DR*
Ong et al. 2000	80	0.903	11.1	
Smith et al. 2002	331	0.963	2.1	7.6
Yaron et al. 2002	27	1.7		
Dugoff et al. 2004	764	1.54	7.85	
Spencer et al.2005b	64	0.844		
Spencer et al. 2008	222	0.772	3.7	14.6
Present study	60	0.956	3.9	

OR, odds ratio; RR, relative risk; DR, detection rate

## Authors Contribution:

**OO **designed the study, did sonographic assessment, collected the data, wrote the manuscript. **IG **collected the data, did sonographic assessment, edited the manuscript. **UK** collected the data, contributed in manuscript writing. **NK **did statistical analysis. **EM **reviewed and edited the manuscript.
